# Effect of anti-sclerostin antibody on orthodontic tooth movement in ovariectomized rats

**DOI:** 10.1186/s40510-024-00544-0

**Published:** 2024-11-25

**Authors:** Hyunna Ahn, Wonse Park, Sung-Hwan Choi, Namki Hong, Jisun Huh, Seoyeon Jung

**Affiliations:** 1 Department of Advanced General Dentistry, Yonsei University College of Dentistry, Seoul, Korea; 2 Human Identification Research Institute, Yonsei University College of Dentistry, Seoul, Korea; 3 Institute for Innovation in Digital Healthcare (IIDH), Yonsei University Health System, Seoul, Korea; 4 Department of Orthodontics, Institute of Craniofacial Deformity, Yonsei University College of Dentistry, Seoul, Korea; 5 Department of Internal Medicine, Severance Hospital, Endocrine Research Institute, Yonsei University College of Medicine, Seoul, Korea; 6 Department of Dental Education, Yonsei University College of Dentistry, Seoul, Korea

**Keywords:** Wnt signaling pathway, Osteoporosis, Bone remodeling, Osteogenesis, Bone biology, Orthodontics

## Abstract

**Background:**

This study aimed to evaluate the effect of systemically administered anti-sclerostin antibodies (Anti-Scl Ab) on orthodontic tooth movements (OTM) in an ovariectomized rat.

**Methods:**

Twenty-four 12-week-old female Sprague-Dawley rats were randomly divided into two groups: (1) ovariectomy (OVX) group, (2) ovariectomy + romosozumab (ROMO) group. OTM was performed 8 weeks after OVX. The ROMO group received subcutaneous injections of romosozumab twice a week, starting two weeks after OVX. Eight weeks after the OVX, an orthodontic force of 50 g was measured and applied by connecting orthodontic elastic bands between the maxillary first molar and a mini-screw to facilitate tooth movement (orthodontic treatment). Subsequently, the three rats were sacrificed on days 5, 7, 10, and 14. The plaster models were scanned to measure the amount of tooth movement. The effects on alveolar bone and periodontal tissues were evaluated through micro-computed tomography (micro-CT) analysis, tartrate-resistant acid phosphatase (TRAP) staining, and immunohistochemistry (IHC) analysis.

**Results:**

The ROMO group showed more tooth movement on day 7 of orthodontic treatment. Conversely, on days 10 and 14, relatively less movement was observed. Analysis of the root furcation area of the maxillary first molars revealed that from the 7th day, BV/TV, Tb.N., Tb.Th. increased, while Tb.Sp. decreased in the ROMO group. More TRAP-positive cells were observed in the compression side of the OVX group, the ROMO group exhibited a marked decrease in the positive expression of the RANKL, OPG, and sclerostin. The OPG/RANKL ratio showed significant differences in expression between the two groups. The ROMO group exhibited a higher OPG/RANKL ratio than the OVX group, and the tension side exhibited a higher OPG/RANKL ratio demonstrating significant differences.

**Conclusion:**

Romosozumab initially accelerated tooth movements, but later decreased tooth movement. As new alveolar bone is formed, the micro-CT parameters are also improved. Osteoclasts, RANKL, OPG, and sclerostin decreased, while the OPG/RANKL ratio became higher.

**Supplementary Information:**

The online version contains supplementary material available at 10.1186/s40510-024-00544-0.

## Background

Orthodontic tooth movement (OTM) is a complex process that involves changes in the teeth and surrounding tissues due to applied mechanical force [1]. Bone resorption in this process is mediated by osteoclasts and cells of the monocyte/macrophage lineage, while osteoclastogenesis is regulated by the receptor activator of nuclear factor kappa B ligand (RANKL) receptor-activator expressed by cells surrounding the tooth root. When orthodontic force is applied, remodeling occurs in the periodontal ligament (PDL) and alveolar bone on the compression side and tension side, which is an important aspect to attention.

The prevalence of orthodontic interventions has expanded beyond the traditional pediatric and adolescent groups. In particular, interesting and demand for orthodontic treatment among middle-aged people is increasing to improve their quality of life and aesthetics [2, 3]. However, people must be cautious with orthodontic treatments as they may have periodontal issues or systemic diseases [4]. Among them, osteoporosis is a disease that cannot be ignored, so caution must be taken during orthodontic treatment.

Osteoporosis elevates the risk of fractures and bone vulnerability in patients, attributed to factors like reduced bone density. This condition is also characterized by diminished bone robustness and compromised bone microarchitecture [5]. In postmenopausal women, the deficiency of estrogen leads to increased bone turnover and more bone resorption than bone formation, increasing the risk of osteoporosis [6]. During orthodontic tooth movement, osteoporosis can cause a potential risk of teeth moving more than intended or relapsing [7]. Recently, among osteoporosis medications, romosozumab has been approved as a dual-effect anabolic agent by the Food and Drug Administration, thereby garnering high interest and expectations.

Romosozumab is a monoclonal antibody that binds to and inhibits the action of sclerostin. It is the first anabolic medication that has a dual action of promoting bone formation and reducing bone resorption by controlling RANKL and OPG [8]. However, to date, there is insufficient evidence regarding the safety and potential side effects of medications required for orthodontic treatments that requires new bone formation. New osteoporosis medications have different mechanisms compared to existing drugs. This indicates a need for further research in this area.

This research aims to fill existing gaps in our understanding by assessing the effects of anti-sclerostin antibodies (Anti-Scl Ab), administered systemically, on OTM in ovariectomized rats. We hypothesized that Anti-Scl Ab, by modulating the expression of RANKL, OPG, and sclerostin, would initially increase OTM, and subsequently decrease it.

## Materials and methods

### Animals

Twenty-four 8-week-old Sprague-Dawley rats (Orient Bio, Seongnam, Korea) were brought in and acclimated for one week. After which ovariectomy was performed at twelve weeks of age in virgin female rats, with a mean weight of 274 g. The selection, management, and surgical protocols were conducted according to the standards approved by the Institutional Animal Care and Use Committee of Yonsei Medical Center, Seoul, Korea (AICUC 2020-0010). The animals were housed in cages with a 12-hour light-dark cycle, at a temperature of 20 °C ± 5 °C, and humidity of 50% ± 10%. They were provided with deionized water and a standard laboratory pellet diet, available ad libitum at all times. Experimental protocols followed ARRIVE guidelines.

### Ovariectomy

All 24 rats underwent surgery at 12 weeks of age. Bilateral ovariectomy was performed under general anesthesia using a combination of intraperitoneal Zoletil^®^ (Tiletamine and zolazepam, 50 mg/ml, 0.6 ml/kg body mass; Virbac lab. Carros, France) and Rompun^®^ (zylazine, 23.32 mg/ml, 0.4 ml/kg body mass; Bayer, Leverkusen, Germany). First, the fur in the surgical area was removed, and the area was disinfected with povidone. Second, a 1 cm incision was made on the dorsal side and separated the musculature using straightened-tip scissors. Third, the ovarian fat pad is pulled out of the incision carefully. Fourth, the ovaries were located, and the incision sites on the oviduct were ligated using sterilized thread. The ovaries were then removed. Finally, the surgical incision site was sutured using Vicryl 4 − 0 sutures (Ethicon, USA). Every surgical implement has been sterilized. After surgery, subcutaneous meloxicam (1 mg/kg, once a day for 3 days; Metacam^®^, Boehringer Ingelheim, Rhein, Germany) and enrofloxacin (10 mg/kg/day, once a day for 3 days; Baytril^®^, Bayer, Germany) were administered. To verify the success of the ovariectomy, the body weight of the rats was measured weekly.

### Medication

The 24 rats were randomly divided into two groups at the animal experimental facility by Animal House staff, who were blinded to the experimental conditions: the ovariectomized (OVX) group and the ovariectomized + romosozumab (ROMO) group. Two weeks after the ovariectomy surgery, the ROMO group received subcutaneous injections of romosozumab (Evenity^®^, Amgen Inc., Thousand Oaks, CA, USA, and UCB Brussels, Belgium) at a dose of 25 mg/kg using a sterile 1 cc syringe, administered twice a week on Mondays and Thursdays. Meanwhile, the OVX group received vehicle injections on the same days. Both groups were treated in this fashion until necropsy.

### Orthodontic tooth movement

**Fig. 1:**
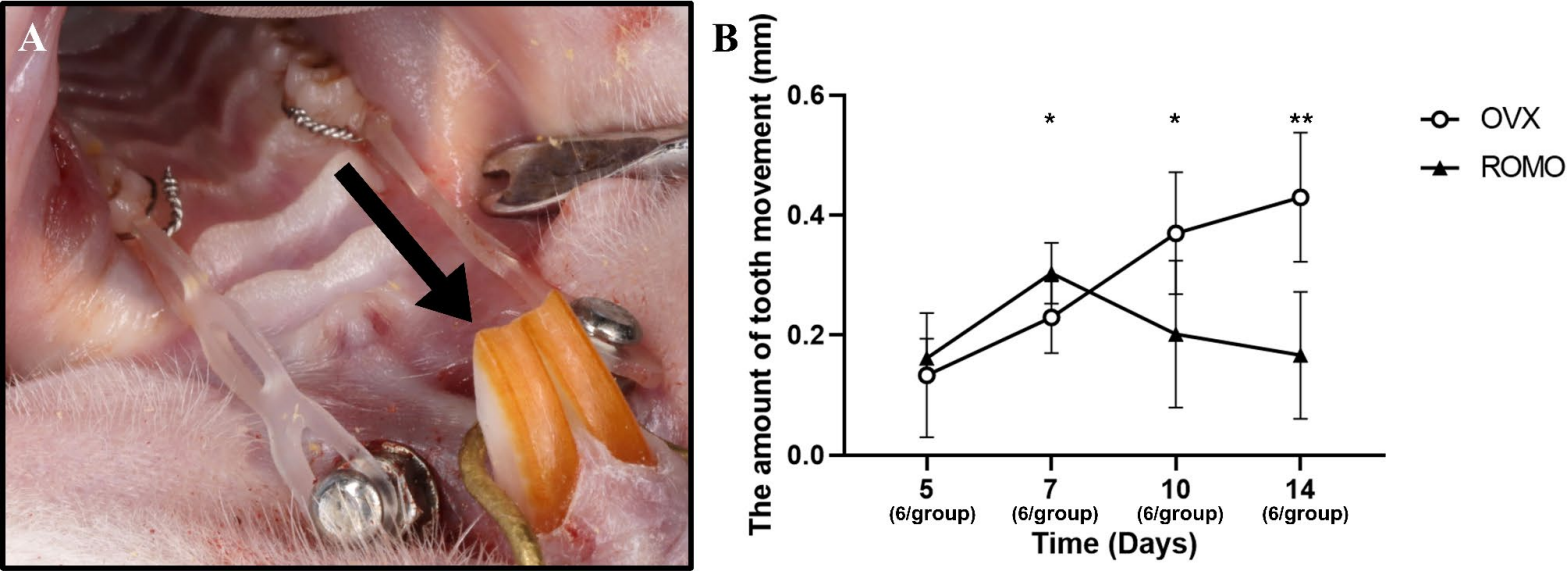
Representative image and amount after orthodontic force. (**A**) Orthodontic tooth movement (OTM) model in rats. The black arrow shows the direction of the mechanical force. (**B**) The distance of OTM on days 5, 7, 10, and 14. Data are presented as the mean ± standard deviation. Independent t-test between OVX and ROMO group at each time point. *: *P* < 0.05; **: *P* < 0.01

In this study, tooth movement was induced in the beginning at rats 8 weeks after ovariectomy. The insert of the mini-screw and connecting power chain was performed under general anesthesia using a combination of intraperitoneal Zoletil^®^ and Rompun^®^. To the anchorage system, a 6 mm mini-screw (Wezendental, Ø 1.6 mm, Seoul, Korea) was inserted into the antero-lateral part of the premaxilla, which is the outer alveolar region of the maxillary incisor (Fig. 1). The sharft (Wezendental, Seoul, Korea) and mini-screw were attached to the screw drive handle (Wezendental, Seoul, Korea) and then directly inserted into the maxillary bone without an incision by turning the handle in a clockwise direction. After inserting the screw into the bone and turning it to seat it firmly, the mini-screw was confirmed to be securely fixed. This created a hole matching the size of the mini-screw. The inserted depth of the mini-screw was approximately 1.6 mm from the cortical bone surface to the bottom tip of the mini-screw. The maxillary first molar and the mini screw were connected using an orthodontic power chain (Generation II, Ormco, Brea, CA, USA). Approximately 50 g of force was measured using a dial tension gauge (DT-50, Techlock Inc, Okaya Japan). This procedure was performed on both the left and right sides of each rat in both groups, resulting in six data points per group (3 rats/group). To maintain a consistent force, the power chain was replaced on days 5 and 10. The ROMO and OVX groups each had 3 rats sacrificed for further analysis on days 5, 7, 10, and 14.

### Distance of orthodontic tooth movement

Before applying the orthodontic power chain, impressions of the maxillary teeth were taken using polyvinylsiloxane impression material (Aquasil Ultra XLV, Dentsply DeTrey GmbH, Konstanz, Germany), and impressions were taken again on days 5, 7, 10, and 14 before sacrifice. To take an impression, an individualized tray for the rats was designed and then printed with a 3D printer. Dental stone (Neo super plumstone, Mutsumi, Japan) was used to create plaster models from the impressions, which were then scanned using the Identica hybrid 3D dental laser scanner (MEDIT T510, Seoul, Korea). The scanned data was provided in standard tessellation language (STL) file format. The OTM distance was measured using the same software (Meshmixer, Autodesk Inc, America). The OTM distance was measured from the mesial height of contour on the maxillary first molar to the distal height of contour on the maxillary third molar. The difference between the post-experiment and pre-experiment measurements was recorded.

### Micro-computed tomography (Micro-CT) analysis

On each sacrifice day, after resecting the maxillary molars, along with adjacent alveolar bone and surrounding tissues, the specimens were fixed in 10% formalin for one week. The power chain was then removed just before micro-CT (Skyscan 1173; SkyScan, Bruker-CT, Kontich, Belgium) scanning, which was subsequently performed under the following conditions: voxel size of 15 μm, voltage of 130 kV, current of 60 µA, and a 1.0 mm aluminum filter. The captured images were then reconstructed and analyzed using CTAn software (Bruker-CT, Kontich, Belgium). A region of interest (ROI) was selected in the furcation area of the maxillary first molar, with dimensions of 400 μm × 400 μm × 500 μm (length × width × thickness, in the sagittal plane). From the extracted data, bone volume/total volume (BV/TV), trabecular thickness (Tb.Th), trabecular number (Tb.N), and trabecular separation (Tb.Sp) were analyzed. Three different regions were selected for each specimen, and the average values were calculated. The definitions of various bone parameters are as follows.

- BV/TV: Represents the ratio of trabecular bone volume to the total bone volume.
- Tb.Th: Measures the average thickness of trabeculae within voxels representing bone using direct three-dimensional (3D) methods.
- Tb.N: Measures the average number of trabeculae per unit length.
- Tb.Sp: Represents the average distance between trabeculae, measured using direct 3D methods.

### Tartrate-resistant acid phosphatase (TRAP) staining

After a one-week fixation in 10% formalin, all samples were subjected to micro-CT scanning. The specimens were then decalcified with 10% EDTA (pH 7), dehydrated, and embedded in paraffin. Sectioning was initiated using a fully automated rotary microtome (RM2255, Leica, Wetzlar, Germany). The cut plane was selected along the axial axis, from the cervical to the apical region of the tooth, and serially sliced into 3–5 μm thickness, ensuring a satisfactory section. These sections were processed for Tartrate-resistant acid phosphatase (TRAP) staining and immunohistochemical (IHC) analysis. Among the five roots of the maxillary first molars in the sample, the mesial root, which is the largest and provides better visualization of the root structure, was selected for histological evaluation.

To evaluate TRAP expression, paraffin blocks of 5 μm thickness were subjected to TRAP staining using a commercial kit (Sigma, St. Louis, MO, USA). Osteoclasts were classified as satisfying the conditions of TRAP-positive, multinucleated cells located along the bone surface. Three randomly selected sections from the mesial root of the maxillary first molar’s compression and tension side were measured and presented as mean ± standard deviation (SD) values.

### Immunohistochemical (IHC) analysis

For IHC staining, paraffin blocks were cut into 3 μm thickness. All IHC staining procedures from deparaffinization to counterstaining were performed using an automated Ventana Discovery XT staining instrument (Ventana Medical Systems, Inc., Tucson, AZ, USA). Mouse anti-RANKL monoclonal antibody (1:6400 dilution; sc-377079, Santa Cruz, USA), rabbit anti-OPG polyclonal antibody (1:400 dilution; ab203061, Abcam, USA), and rabbit anti-sclerostin polyclonal antibody (1:400 dilution; ab85799, Abcam, USA) were used as primary antibodies. Secondary antibodies were applied according to the manufacturer’s recommendations. Subsequently, the sections were visualized using a diaminobenzidine (DAB) coloration kit and counterstained with hematoxylin. Their presence was observed in the mesial root of each maxillary first molar, as indicated by a brownish-yellow color. The IHC staining intensity was measured using the IHC profiler plugin in ImageJ software (version 1.53, National Institutes of Health, USA) after magnifying the ROI in the gingival area by 200x. The plugin’s colour deconvolution method was used to digitally separate DAB and hematoxylin nuclear staining and calculate their respective contribution. After deconvolution, the DAB image was converted to an 8-bit format, and a threshold value was set to consider black pixels as positive DAB staining. The stained RANKL, OPG, and sclerostin were quantified based on the values of black pixels in the respective ROIs and the overall mean areas were compared between groups.

All histological stained slides were scanned using the Aperio AT2 image-capturing device (Leica Biosystems, Wetzlar, Germany), and evaluated at x200 and x400 magnification using Imagescope software 12.3 (Aperio Technologies, Vista, CA, USA). The stained slides were analyzed by a single-blinded researcher to avoid procedure differences that may affect experimental results between operators.

### Statistical analysis

This study was analyzed using SPSS Version 23.0 (Statistical Package for the Social Sciences, IBM, USA) at a 95% confidence level. All data were presented as mean ± SD. All of the data were first subjected to two-way ANOVA analysis to compare mean values between the group, time, and the group x time interaction. When ANOVA indicated a difference between factors, post hoc comparisons between OVX and ROMO groups at each time point were performed using an independent t-test or Mann-Whitney test. An independent t-test was used to analyze the distance of OTM and micro-CT parameters. For analyzing the body weight, the number of TRAP-positive cells, and the expression levels of RANKL, osteoprotegerin, sclerostin, and the ratio of OPG and RANKL, the Mann-Whitney test was employed.

## Results

### The mean body weight of the rats

Supplementary Table 1 shows the rat weight (mean ± SD) of all groups. In general, animal’s body weight increases with time. In the OVX and ROMO groups, the average body weight increased after OVX and before OTM.

### The amount of tooth movement

The amounts of OTM are shown in Fig. 1. The amounts of tooth movement were different between groups. The differences in means between groups, over time, and the group x time interaction were all statistically significant (*p* = 0.009, *p* = 0.005, and *p* < 0.001, respectively). Post hoc analysis comparing the OVX group and the ROMO group showed that a statistically significant difference was observed on days 7, 10, and 14. The OVX group showed a statistically significant difference between day 5 and day 10, as well as between day 5 and day 14. Additionally, there was a statistically significant difference between day 7 and day 14. The ROMO group showed a statistically significant difference between day 5 and day 7, as well as between day 7 and day 14.

### Micro-computed tomographic (Micro-CT) analysis

**Fig. 2:**
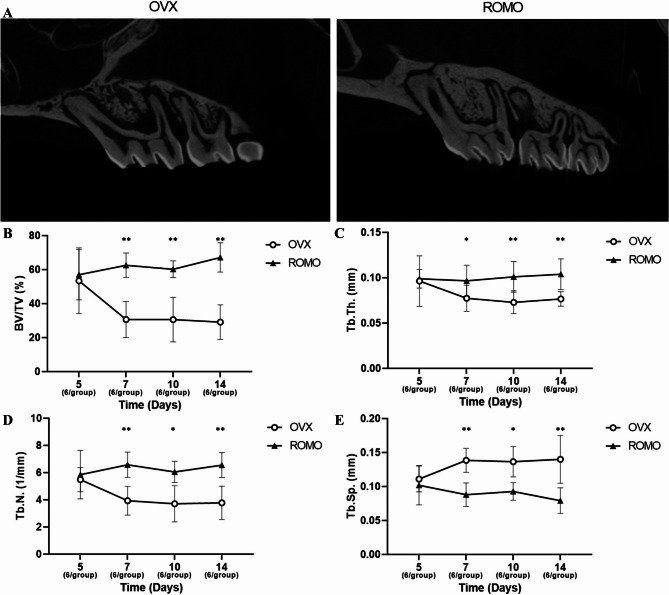
Micro-CT analysis of furcation area of the maxillary first molar. (**A**) Micro-CT scan of the maxillae in the control and experimental groups, (**B**) Micro-CT anlalysis of BV/TV. (**C**) Micro-CT analysis of Tb.Th. (**D**) Micro-CT analysis of Tb.N. (**E**) Micro-CT analysis of Tb.Sp. The following numbers of rats were measured: Day 5 (3 rats/group); Day 7 (3 rats/group); Day 10 (3 rats/group); Day 14 (3 rats/group). Independent t-test between OVX and ROMO group at each time point. *: *P* < 0.05; **: *P* < 0.01

The micro-CT results showed differences in bone parameters between the groups, over time, and group x time interaction. For BV/TV values, both the difference between groups and the group x time interaction were statistically significant (*p* < 0.001 and *p* = 0.005, respectively). For Tb.Th. values, the difference between groups was statistically significant (*p* < 0.001). For Tb.N. values, the difference between groups and group x time interaction were statistically significant (*p* < 0.001 and *p* = 0.049, respectively). For Tb.Sp. values, the difference between groups and group x time interaction were statistically significant (*p* < 0.001 and *p* = 0.044, respectively). Post hoc analysis of the furcation area of the maxillary first molar showed romosozumab increased BV/TV, BV/Th, and Tb.N, and decreased Tb.Sp, with significant differences observed in all parameters. (Fig. 2).

### Histological analysis

**Fig. 3:**
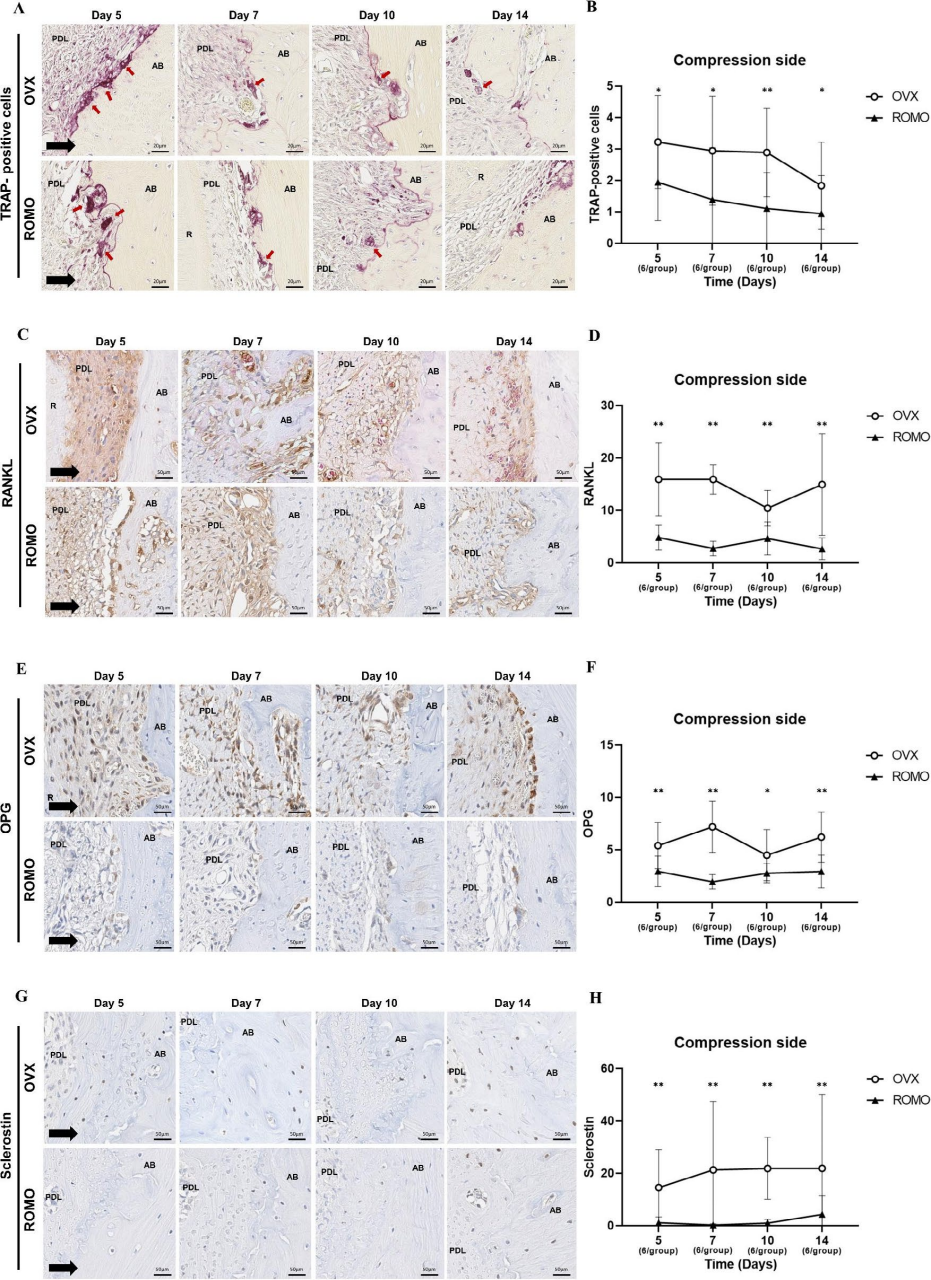
Histological analysis of TRAP, RANKL, OPG, and Sclerostin at compression side. (**A**) The TRAP staining on the compression side. (**B**) The number of TRAP-positive osteoclasts on the compression side. (**C**) The IHC staining of RANKL at the compression side. (**D**) The positive expression of RANKL. (**E**) The IHC staining of OPG at the compression side. (**F**) The positive expression of OPG. (**G**) The IHC staining of sclerostin at the compression side. (**H**) The positive expression of sclerostin. AB, alveolar bone; PDL, periodontal ligament; R, root; D, dentin. The following numbers of rats were measured: Day 5 (3 rats/group); Day 7 (3 rats/group); Day 10 (3 rats/group); Day 14 (3 rats/group). Mann-Whitney test between OVX and ROMO group at each time point. *: *P* < 0.05; **: *P* < 0.01

**Fig. 4:**
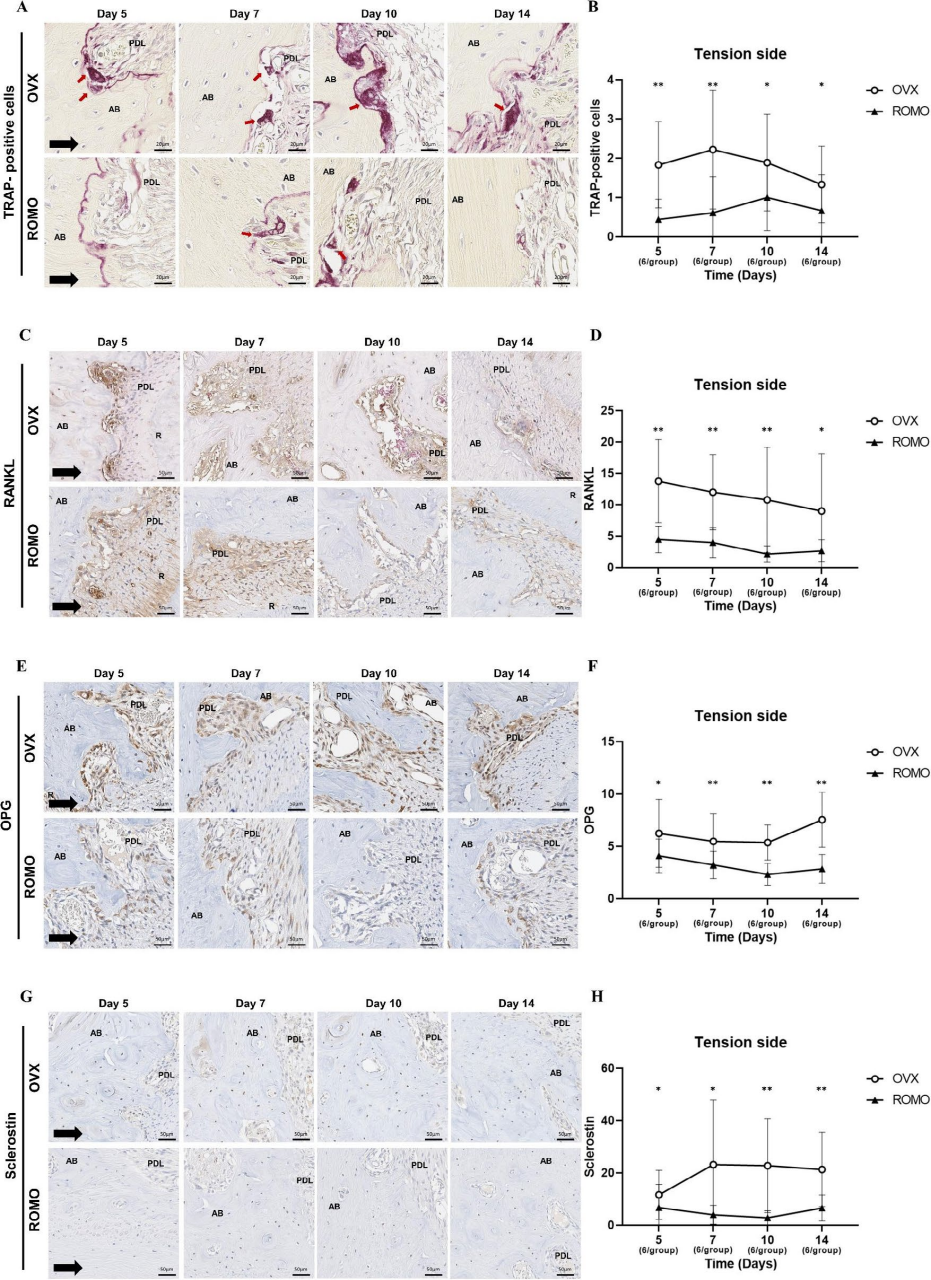
Histological analysis of TRAP, RANKL, OPG, and Sclerostin at tension side. (**A**) The TRAP staining on the tension side. (**B**) The number of TRAP-positive osteoclasts on the tension side. (**C**) The IHC staining of RANKL at the tension side. (**D**) The positive expression of RANKL. (**E**) The IHC staining of OPG at the tension side. (**F**) The positive expression of OPG. (**G**) The IHC staining of sclerostin at the tension side. (**H**) The positive expression of sclerostin. AB, alveolar bone; PDL, periodontal ligament; R, root; D, dentin. The following numbers of rats were measured: Day 5 (3 rats/group); Day 7 (3 rats/group); Day 10 (3 rats/group); Day 14 (3 rats/group). Mann-Whitney test between OVX and ROMO group at each time point. *: *P* < 0.05; **: *P* < 0.01

The compression side can be seen in Fig. 3 and the tension side can be seen in Fig. 4. For TRAP-positive cells, the differences between groups and over time were statistically significant (*p* < 0.001 and *p* = 0.005, respectively). Post hoc analysis showed that the OVX group showed a higher number of TRAP-positive cells on both the compression and tension sides compared to the ROMO group, indicating significant differences between the groups at all time points.

RANKL and OPG were observed in the PDL cells. Immunohistochemical analysis for RANKL expression showed that the difference between groups and group x time interaction were statistically significant (*p* < 0.001 and *p* = 0.006, respectively). Post hoc analysis showed that the ROMO group showed lower positive expression of RANKL on both the compression and tension sides compared to the OVX group, demonstrating significant differences between the groups at all time points. For OPG expression, the difference between groups and group x time interaction were statistically significant (*p* < 0.001 and *p* < 0.001, respectively). Post hoc analysis showed that the ROMO group also showed lower levels of OPG positive expression on both sides, with significant differences at all time points. Sclerostin was observed in the osteocytes of the alveolar bone. For sclerostin expression, the difference between groups was statistically significant (*p* < 0.001). Post hoc analysis showed that a decrease in sclerostin was observed at all time points in rats administered with romosozumab. After the administration of romosozumab, there was a marked decrease in the positive expression of RANKL, osteoprotegerin, and sclerostin on both the compression and tension sides.

**Fig. 5:**
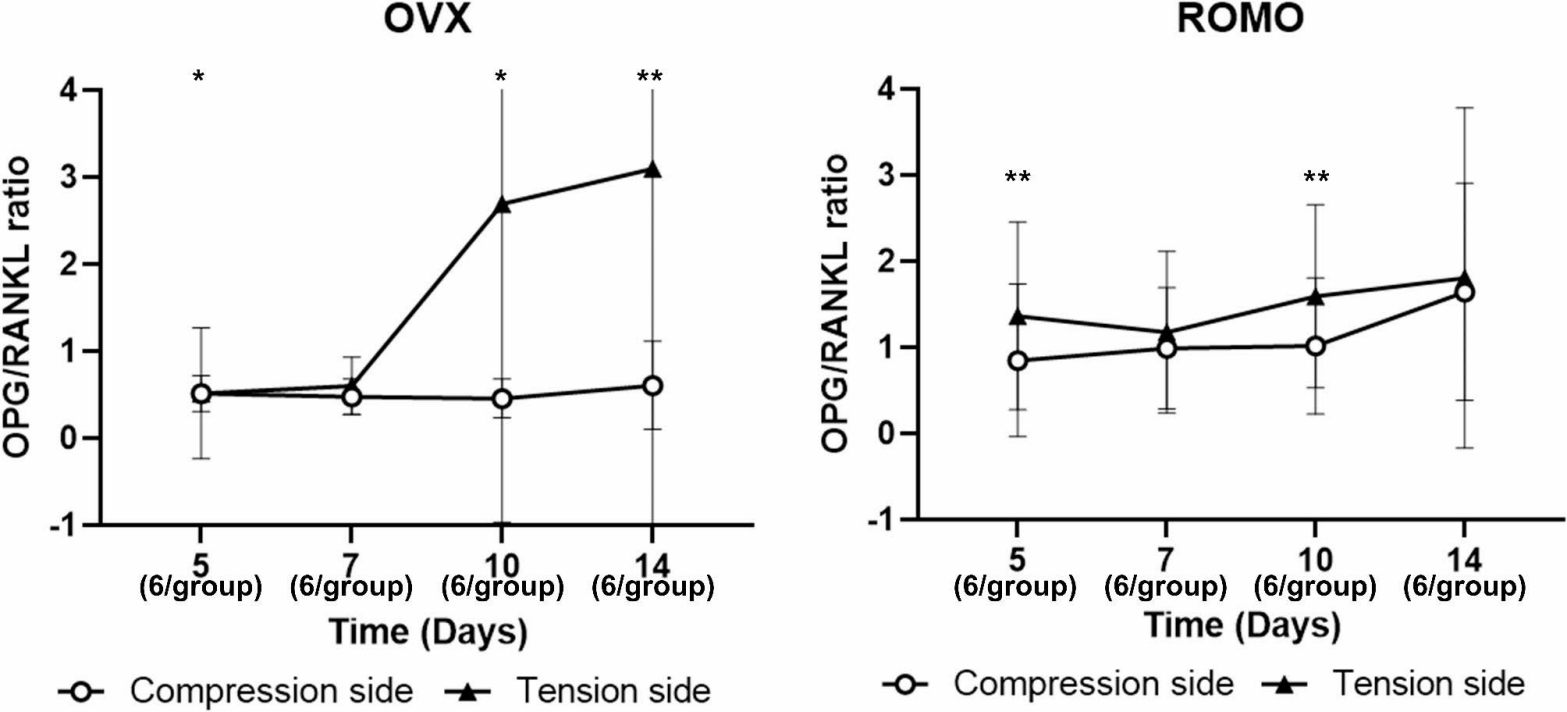
The ratio of OPG/RANKL according to group. OVX (ovariectomy group), ROMO (ovariectomy + romosozumab group). The following numbers of rats were measured: Day 5 (3 rats/group); Day 7 (3 rats/group); Day 10 (3 rats/group); Day 14 (3 rats/group). Mann-Whitney test between OVX and ROMO group at each time point. *: *P* < 0.05; **: *P* < 0.01

From an immunohistochemical perspective, RANKL/OPG ratio showed that the difference between groups and over time were statistically significant (*p* < 0.001 and *p* = 0.045, respectively). Post hoc analysis showed that the OPG/RANKL ratio demonstrated marked expression differences between the two groups (Fig. 5). On the compression side in remodeling sites, the ROMO group exhibited a higher OPG/RANKL ratio than the OVX group, demonstrating statistically significant differences at all time points. On the tension side in remodeling sites, the ROMO group showed a higher OPG/RANKL ratio on days 5 and 7. A difference between the compression and tension sides was also observed. According to quantitative data, the OPG/RANKL ratio was higher on the tension side than on the compression side in both the OVX and ROMO groups.

## Discussion

We used an Anti-Scl Ab to study the effects of sclerostin inhibition in OVX rats. The basis of orthodontic treatment is that when appropriate force is delivered to the teeth, they move through the PDL and alveolar bone. Deficiency of estrogen caused by ovariectomy during OTM shortens the lifespan of osteoblasts and osteocytes and impairs their function, and the lifespan of osteoclasts is extended and differentiated, leading to active resorption activity [9, 10]. As a result, the amount of OTM in the OVX group showed an increasing tendency at each measurement point during the experiment. In contrast, in the ROMO group, the amount of OTM showed an increasing tendency until day 7, and then, a decreasing tendency was observed from day 10. Previous research has shown that in ovariectomized rats, tooth movement occurs more rapidly compared to non-ovariectomized rats, with an increase in osteoclast formation, which supports the results of tooth movement observed in this study [11–13]. The experimental period of this study was chosen to observe the bone remodeling process and evaluate the duration of the application’s effects during the accelerated orthodontics process. On day 5, differences in RANKL and OPG indicated the initiation of bone remodeling, but no significant differences were observed in tooth movement and bone parameters, suggesting that the effects of the drug had yet to manifest. However, from day 7 onward, changes in tooth movement and bone parameters were observed, indicating the onset of active bone remodeling. By day 10, the bone parameters were maintained, the OPG/RANKL ratio increased, and tooth movement showed a decreasing tendency.

Due to the different mechanisms of action, osteoporosis medications are expected to show varying outcomes on orthodontic tooth movement. Therefore, this study focused on investigating the effects of romosozumab, a bone formation stimulant. In the case of osteoporosis patients, drugs are typically administered after the onset of osteoporosis for treatment purposes. However, in our study, the goal was not to treat osteoporosis but to observe the effects of osteoporosis-related conditions on orthodontic treatment. Therefore, the experimental design was based on previous research in this field [14, 15]. Previous studies demonstrated that sclerostin injected rats exhibited accelerated tooth movement, which is similar to the findings of this study [16, 17]. Previous research has reported that antiresorptive drug reduced the amount of movement [14, 18, 19], while PTH, anabolic agent, increases it [20–22]. However, romosozumab, an anabolic agent, has shown a different effect on tooth movement compared to other drugs. Our study demonstrated for the first time that romosozumab regulates tooth movement by temporarily accelerating and then decreasing it.

Micro-CT has been used to assess the three-dimensional microstructure of alveolar bone, allowing for the evaluation of trabecular and cortical bone morphology in animal specimens during the OTM process. Micro-CT analysis revealed that BV/TV, Tb.Th, and Tb.N increased and Tb.Sp decreased in the group that underwent OTM after romosozumab administration compared to the group that did not, showing differences in all parameters. During 14 days of orthodontic treatment, ROMO group showed significantly improved trabecular bone parameters. However, this effect was not observed on day 5, which suggested postponed protection of alveolar bone mass by romosozumab. Bone parameters are adjusted because Anti-Scl Ab stimulates bone turnover and normalizes bone mass, thereby maintaining or improving bone strength and bone quality. Existing animal studies have shown that administration of sclerostin antibody improved bone strength and bone mass [23, 24]. These results are similar to the results of this study observed in the furcation area. Similar to the findings observed in the remodeling area of this study, the results indicate that administering romosozumab can suppress alveolar bone loss or promote the formation of alveolar bone. The administration of Anti-Scl Ab not only reduced BV/TV in the furcation area, but also improved bone strength and mass.

RANKL, a ligand related to tumor necrosis factor (TNF), is expressed on the surface of osteoclast precursor cells, and binds to its receptor, RANK, to rapidly differentiate hematopoietic osteoclast precursors to function as mature osteoclasts. OPG, a member of the TNF family, is also a decoy receptor produced from osteoblasts and binds to RANKL, preventing RANK from binding to RANKL [25]. In other words, when RANKL expression increases and OPG expression decreases, it is favorable for osteoclast formation and bone resorption progresses rapidly, while when RANLK expression decreases and the rate of OPG expression increases, bone formation is promoted [26]. Sclerostin binds to the LRP5/6 complex and inhibits Wnt/β-catenin signaling, which is crucial for bone formation. The activation of the Wnt signaling pathway promotes the expression of OPG, but sclerostin reduces OPG and induces the action of RANKL, thereby promoting bone resorption. Romosozumab inhibits sclerostin, reducing the RANKL/OPG ratio, which in turn suppresses bone resorption while simultaneously promoting bone formation through its dual action. Because osteoclasts are stimulated through the RANKL, RANK, and OPG pathways for differentiation and activity, the ratio of OPG expression to RANKL expression is related to the formation of osteoclastogenesis [27]. In this study, not only TRAP-positive cells but also RANKL expression levels decreased in a similar manner, and this change is similar to previous studies showing that administration of anti-sclerostin regulates the ratio of RANKL and OPG expression in osteocytes [16].

Our study found that romosozumab downregulated sclerostin, RANKL, and OPG and increased the OPG/RANKL ratio. Sclesrostin protein accelerates OTM while simultaneously upregulating RANKL and downregulating OPG [16]. It has been shown that inhibition of sclerostin affects osteoclast and bone resorption in animals with induced osteoporosis [5, 8]. It was confirmed that TRAP-positive cells were downregulated in the ROMO group due to the effect of romosozumab on osteoclasts. Downregulation of osteoclasts can affect the amount of tooth movement, and reduced osteoclast activity can be said to reduce bone resorption and consequently reduce tooth movement. Unlike the change in TRAP-positive cells, it was observed that tooth movement has not continuously decreased. However, since tooth movement is a combination of not only osteoclast but also various biological processes, further research on the regulatory mechanisms of romosozumab seems necessary.

Romosozumab promotes bone formation through a modeling-based mechanism and increases bone mass, trabecular, and cortical bone volume. What bone modeling and remodeling have in common is that they are both influenced by osteocytes, osteoblasts, and osteoclasts. The difference between the two is that in bone modeling, resorption and formation activities do not occur together, but bone remodeling is a local process in which new bone is formed after resorption and old bone is replaced by new bone [28]. Although this study only carried out histological analysis, the high OPG/RANKL ratio observed in the ROMO group of this study. That may suggest that romosozumab promotes bone formation and inhibits bone resorption. However, it is difficult to assert the changes solely to remodeling, as the OPG/RANKL ratio is influenced not only by bone remodeling but also by bone modeling processes.

The limitation of this study is that it did not include a comparison with rats that had not undergone ovariectomy or a male cohort. This signifies that there was no comparison made with a normal rat. Although the effectiveness of romosozumab has been proven in ovariectomized rats, it is unclear whether the value falls within the normal range when compared to normal rats, so follow-up research on this appears to be necessary. A second limitation of this study is the use of 3 rats/group on days 5, 7, 10, and 14.

## Conclusion

The administration of romosozumab initially accelerated tooth movement temporarily but later inhibited tooth movement. In the ROMO group, bone remodeling markers and sclerostin decreased, but the OPG/RANKL ratio became higher. These observations suggest that changes in bone metabolism can regulate tooth movement, and therefore osteoporotic patients should be taken into consideration when they receive orthodontic treatment.

## Electronic supplementary material

Below is the link to the electronic supplementary material.

Supplementary Material 1

## Data Availability

No datasets were generated or analysed during the current study.

## Abbreviations

OTM: Orthodontic tooth movements
RANKL: Receptor activator of nuclear factor kappa B ligand
PDL: Periodontal ligament
Anti-Scl Ab: Anti-sclerostin antibodies
OVX: Ovariectomy
ROMO: Ovariectomy + romosozumab
BV/TV: Bone volume/total volume
Tb.Th: Trabecular thickness
TbN: Trabecular number
Tb.Sp: Trabecular separation
TRAP: Tartrate-resistant acid phosphatase
IHC: Immunohistochemistry
DAB: Diaminobenzidine
SD: Standard deviation
Micro-CT: Micro-computed tomography
TNF: Tumor necrosis factor
