# Correction to: Effect of anti-sclerostin antibody on orthodontic tooth movement in ovariectomized rats

**DOI:** 10.1186/s40510-024-00552-0

**Published:** 2025-01-03

**Authors:** Hyunna Ahn, Wonse Park, Sung-Hwan Choi, Namki Hong, Jisun Huh, Seoyeon Jung

**Affiliations:** 1 Department of Advanced General Dentistry, Yonsei University College of Dentistry, Seoul, Korea; 2 Human Identification Research Institute, Yonsei University College of Dentistry, Seoul, Korea; 3 Institute for Innovation in Digital Healthcare (IIDH), Yonsei University Health System, Seoul, Korea; 4 Department of Orthodontics, Institute of Craniofacial Deformity, Yonsei University College of Dentistry, Seoul, Korea; 5 Department of Internal Medicine, Severance Hospital, Endocrine Research Institute, Yonsei University College of Medicine, Seoul, Korea; 6 Department of Dental Education, Yonsei University College of Dentistry, Seoul, Korea

**Correction to: Prog Orthod. 25, 45 (2024)**

10.1186/s40510-024-00544-0

Following publication of the original article [1], the authors identified that the equal contribution was missing.

^†^Hyunna Ahn and Wonse Park contributed equally to this work.

The equal contribution has been updated above and the original article [1] has been corrected.

## References

[1] Ahn H, Park W, Choi SH, et al. Effect of anti-sclerostin antibody on orthodontic tooth movement in ovariectomized rats. Prog Orthod. 2024;25:45. 10.1186/s40510-024-00544-0. 39581932 10.1186/s40510-024-00544-0 PMC11586325

