# Supplementary material for: Effect of anti-sclerostin antibody on orthodontic tooth movement in ovariectomized rats
Source: Prog Orthod. 2024 Nov 25;25:45. doi: 10.1186/s40510-024-00544-0 (PMC11586325; doi:10.1186/s40510-024-00544-0)
Supplement: Supplementary file 1 — Supplementary Material 1 [file 40510_2024_544_MOESM1_ESM.docx]

**Supplementary Table 1** Comparison of body weight between the OVX and ROMO groups according to age

| **Group** | **0 w** | **1 w** | **2 w** | **3 w** | **4 w *** | **5 w** | **6 w** | **7 w** | **8 w *** | **9 w** | **10 w** |
| --- | --- | --- | --- | --- | --- | --- | --- | --- | --- | --- | --- |
| **OVX** | 265 ± 9 | 292 ± 14 | 305 ± 14 | 336 ± 16 | 352 ± 15 | 366 ± 17 | 363 ± 15 | 372 ± 16 | 377 ± 17 | 344 ± 14 | 351 ± 8 |
| **ROMO** | 262 ± 14 | 277 ± 21 | 295 ± 24 | 321 ± 23 | 333 ± 24 | 342 ± 22 | 349 ± 24 | 355 ± 26 | 346 ± 35 | 332 ± 25 | 322 ±30 |

Mean weight (g) of animals per group. Mann-Whitney test between OVX and ROMO group at each time point. * *P* < .05.
